# Preparation of CS-LS/AgNPs Composites and Photocatalytic Degradation of Dyes

**DOI:** 10.3390/ma17051214

**Published:** 2024-03-06

**Authors:** Jiabao Wu, Xinpeng Chen, Aijing Li, Tieling Xing, Guoqiang Chen

**Affiliations:** Discharge Reduction and Cleaner Production (ERC), Jiangsu Engineering Research Center of Textile Dyeing and Printing for Energy Conservation, College of Textile and Clothing Engineering, Soochow University, Suzhou 215123, China; yzwujiabao@163.com (J.W.); chenxinpeng112@163.com (X.C.); liaijing913@163.com (A.L.); chenguojiang@suda.edu.cn (G.C.)

**Keywords:** chitosan, sodium lignosulfonate, nanosilver, degradation, photocatalysis, dye wastewater

## Abstract

Synthetic dyes are prone to water pollution during use, jeopardizing biodiversity and human health. This study aimed to investigate the adsorption and photocatalytic assist potential of sodium lignosulfonate (LS) in in situ reduced silver nanoparticles (AgNPs) and chitosan (CS)-loaded silver nanoparticles (CS-LS/AgNPs) as adsorbents for Rhodamine B (RhB). The AgNPs were synthesized by doping LS on the surface of chitosan for modification. Fourier transform infrared (FT-IR) spectrometry, energy-dispersive spectroscopy (EDS), scanning electron microscopy (SEM), X-ray diffraction (XRD), and X-ray photoelectron spectroscopy (XPS) were used to confirm the synthesis of nanomaterials. The adsorption and photocatalytic removal experiments of RhB were carried out under optimal conditions (initial dye concentration of 20 mg/L, adsorbent dosage of 0.02 g, time of 60 min, and UV power of 250 W), and the kinetics of dye degradation was also investigated, which showed that the removal rate of RhB by AgNPs photocatalysis can reach 55%. The results indicated that LS was highly effective as a reducing agent for the large-scale production of metal nanoparticles and can be used for dye decolorization. This work provides a new catalyst for the effective removal of dye from wastewater, and can achieve high-value applications of chitosan and lignin.

## 1. Introduction

Synthetic dyes are widely used in different industries, including textile, leather, food, and paper. The industrial wastewater produced after industrial production is not effectively purified and is commonly discharged into the ocean or permeates the soil [1]. These dyes are difficult to biodegrade due to their complex chemical nature and structure, and their dispersion in water is persistent [2,3]. The discharge of these dyes into water bodies is harmful to biodiversity and hurts human health [4,5,6]. Therefore, it is necessary to provide effective methods for removing dyes from wastewater. Although there are many methods for treating dye wastewater, most of them are complex. Relatively, adsorption has been found to be a more effective method [7,8,9], and, as reported elsewhere, it has emerged as a promising and versatile technique for water purification.

Precious metal nanoparticles such as nanosilver exhibit a surface plasmon resonance effect located within the visible range, where surface valence electrons generate collective oscillations under the action of external fields such as light. During this process, electron–hole pairs (e^−^–h^+^) are generated on the surface of nanosilver. e^−^ and h+ are involved in the surface catalytic reactions through electron transfer with oxygen in water as well as water to generate superoxide anion radicals (·O_2_^−^) and hydroxyl radicals (·OH), respectively, which can significantly enhance visible light absorption [10,11], thus achieving the purpose of organic treatment [12,13,14]. The synthesis of nanosilver mainly includes physical, chemical, and biological methods. In recent years, due to the call for green chemistry and energy saving, green preparation methods for nanosilver have emerged. Researchers used lemon extract, grape seed, and Ganoderma lucidum leaf extract as reducing agents for the green synthesis of nanosilver particles, and the synthesized nanosilver has suitable and stable particle size [15,16,17]. Compared to other biomaterials, plant extracts have a wider range of sources and are easier to use, making them suitable for large-scale nanomaterials preparation. Lignin is the second most abundant renewable biomass in nature, second only to cellulose, and exists along with cellulose and hemicellulose in woody biomass [18]. Lignin and its derivatives contain a variety of functional groups (carbonyl, hydroxyl, methoxy, etc.) [19], which gives them potential as adsorbent materials with functional groups that can reduce silver nitrate in situ and provide attachment sites for silver nanoparticles. As green and low-cost additives, lignin and its derivatives also have sufficient impact resistance and tensile properties to prevent the collapse of material structure and improve the adaptability of material [20]. Chitosan is a polysaccharide obtained from the deacetylation of chitin and belongs to an environmentally friendly and efficient adsorbent. It contains several organic functional groups that can participate in the adsorption process. However, unmodified chitosan has limited applications due to its low chemical stability and insufficient adsorption capacity. Therefore, different chitosan-based biomaterials have been synthesized [21,22,23].

In this work, a novel photocatalyst (CS-LS/AgNPs) was prepared to effectively remove organic dyes from water by introducing sodium lignosulfonate (LS) into chitosan (CS) and crosslinking it to form composite particles, and then loading silver nanoparticles (AgNPs) onto the composite particles through in situ reduction. The samples were characterized by scanning electron microscopy (SEM), energy dispersive spectroscopy (EDS), Fourier transform infrared (FT-IR) spectrometry, X-ray diffraction (XRD), and X-ray photoelectron spectroscopy (XPS). The effects of CS dosage, LS: silver nitrate (SN) content ratio, and reaction time on the photocatalytic degradation performance of Rhodamine B (RhB) were investigated in detail. The adsorption kinetics were investigated to understand the catalytic mechanism. In addition, the photocatalytic degradation mechanism of CS-LS/AgNPs on RhB was further discussed based on DMPO quenching experiments and ESR experiments. Finally, the regeneration performance of CS-LS/AgNPs was investigated based on its recovery potential. This study suggests that LS is a highly effective reducing agent for large-scale production of metal nanoparticles and can be used for dye decolorization. Additionally, it provides a new catalyst for the efficient removal of dye from wastewater, allowing for high-value applications of chitosan and lignin.

## 2. Materials and Methods

### 2.1. Materials

**Figure 1:**
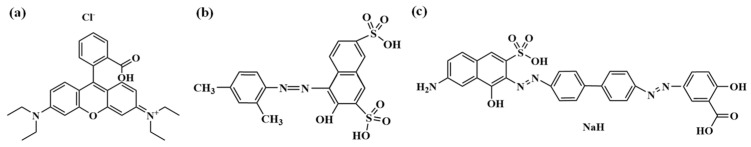
Structural of (**a**) Rhodamine B (RhB), (**b**) Telon Red A2R, and (**c**) Direct Dark Brown ME.

Sodium ligninsulfonate, chitosan (degree of deacetylation: 95%), glutaraldehyde, isopropyl alcohol (IPA), ethylene diamine tetraacetic acid (EDTA), p-benzoquinone (BQ), Rhodamine B, and Direct Dark Brown ME were purchased from Shanghai Aladdin Biochemical Technology Co., (Shanghai, China). Telon Red A2R was purchased from DyStar China (the structure of the three dyes are shown in Figure 1). Silver nitrate was bought from Shanghai Institute of Fine Chemical Materials. Hydrochloric acid was supplied by Shanghai Lingfeng Chemical Reagent Co., (Shanghai, China). All chemicals were used without further treatment. Deionized water prepared in the laboratory was used for all experiments.

### 2.2. Preparation Methods

**Figure 2:**
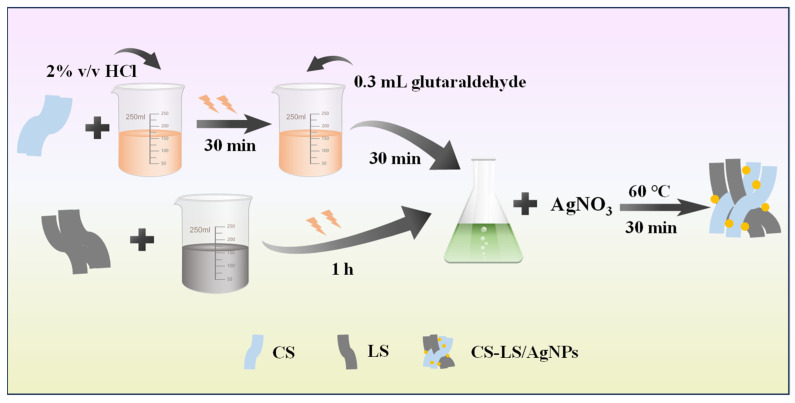
Synthesis pathway of CS-LS/AgNPs.

The synthetic route of CS-LS/AgNPs composites is schematically shown in Figure 2. First, 0.3 g of chitosan was dissolved in 25 mL of dilute hydrochloric acid (2% *v*/*v*), sonicated for 30 min, and then 0.3 mL of glutaraldehyde was added and stirred for 30 min, which was recorded as A. Meanwhile, 5.34 g of LS was sonicated in 20 mL of deionized water for 1 h to dissolve LS, which was recorded as B. CS-LS/AgNPs composites were obtained by mixing A and B homogeneously and treating with a ready-made AgNO_3_ solution (0.1 mol/L) to grow Ag nanoparticles in situ on the surface of CS. CS-LS/AgNPs composites were generated through the redox reaction between silver ions and LS.

### 2.3. Material Characterization

The pristine and modified CS and CS-LS/AgNPs were analyzed by FTIR spectrometer using a Nicolet-5700 FTIR spectrometer (Thermo Scientific, Waltham, MA, USA). The samples to be tested were ground into very fine powder and sampled with potassium bromide. The scanning range was 500–4000 cm^−1^ and the number of scans was 32. The samples were pressed before FTIR analysis and the background spectra were recorded using a potassium bromide powder press. The surface morphology of all products (including CS, CS-LS, and CS-LS/AgNPs) was analyzed using a benchtop scanning electron microscope (Zeiss Gemini 300, Oberkochen, Germany) at an accelerating voltage of 15 kV. EDS was performed using a Bruckner axis EDS analyzer with SEM installed (Brueckner Group China Co., Ltd., Suzhou, China). The crystal phase composition was also characterized by powder XRD (Rigaku SmartLab SE, Tokyo, Japan) using Cu Kα radiation (1.5418 Å). Narrow scan spectra of C 1s, N 1s, and Ag 3d were fitted using XPS (Thermo Scientific K-Alpha, Waltham, MA, USA) as well as Avantage XPS software (Thermo Avantage v5.948). The Brunauer–Emmett–Teller (BET) surface areas of CS-LS/AgNPs were obtained by an ASAP 2460 Surface Area and Porosimetry Analyzer (Micromeritics, Shanghai, China). The UV absorption spectra of RhB solutions were obtained at 554 nm on a UV–Vis spectrophotometer (TU1900, Beijing, China).

### 2.4. Adsorption Experiments

The adsorption experiments of CS-LS/AgNPs composites on RhB dye solution were carried out according to the following procedure. First, 0.02 g CS-LS/AgNPs were added to 20 mg/L RhB (20 mL) solution with stirring. The reaction was performed under dark conditions for 60 min. The change in RhB dye concentration under light-free conditions was quantified by UV–Vis spectra and standard curves of absorbance versus RhB concentration.

### 2.5. Photocatalytic Experiments

The catalytic performance of the synthesized CS-LS/AgNPs composites was investigated by simulating typical pollutants with RhB solution. In the photocatalytic experiments, 20 mg of prepared CS-LS/AgNPs were added to a beaker containing 20 mL of RhB (20 mg/L) solution. The catalytic process was monitored by measuring the change in absorbance of the UV–Vis spectrum at 554 nm (time of 60 min and UV power of 250 W), and the residual concentration of RhB in the solution was calculated from the standard absorbance curve of absorbance versus RhB concentration.

The catalytic degradation percentage was calculated as (C_0_ − C_t_)/C_0_, where C_0_ is the initial concentration of RhB and Ct is the concentration at reaction time t. The recoverability and reusability of CS-LS/AgNPs were also evaluated. The recovered catalysts were fully washed with ethanol and deionized water before the next catalytic test.

## 3. Results

### 3.1. Characterization of CS-LS/AgNPs Composites

**Figure 3:**
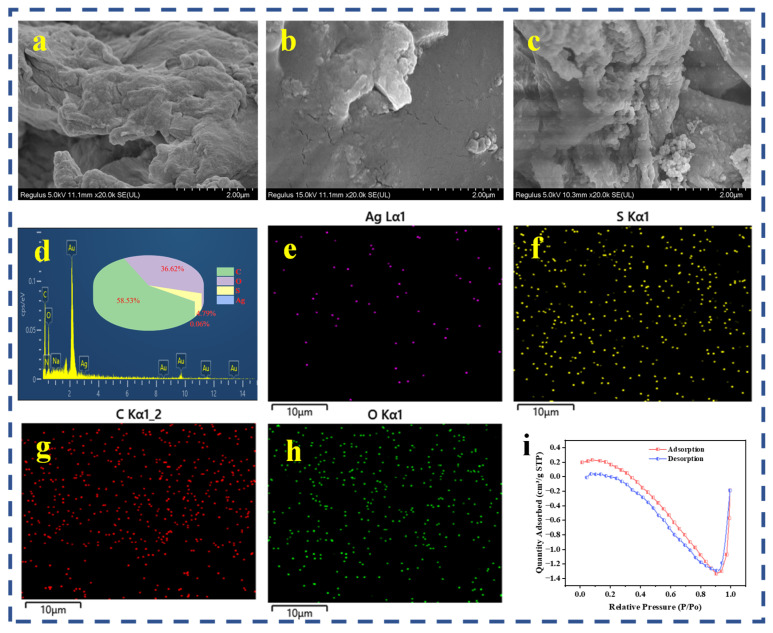
SEM analysis of CS-LS/AgNPs: (**a**) CS, (**b**) CS-LS, (**c**) CS-LS/AgNPs, (**d**) EDS analysis, (**e**–**h**) elemental mapping of CS-LS/AgNPs, and (**i**) N_2_ sorption isotherm of CS-LS/AgNPs.

The morphologies of CS, CS-LS, and CS-LS/AgNPs were characterized using scanning electron microscopy. SEM images of CS, CS-LS, and CS-LS/AgNPs are shown in Figure 3a–c, respectively. It can be clearly observed that CS itself had surface grooves and intertwined structures with smooth surfaces (Figure 3a). Compared with CS, the surface morphology of CS-LS (Figure 3b) is flatter and more coherent, while many bumps are formed and the structure is greatly changed, indicating that CS formed a tighter bond with LS, and LS was successfully loaded onto the CS surface. After silver nitrate treatment, there are obvious particles loading on the CS surface, which is judged to be nanosilver. At the same time, due to the cellulose properties of CS as well as LS, the silver nanoparticles can be uniformly distributed in large amounts on the surface of CS (Figure 3c). To confirm the elemental changes in the chemical composition before and after the preparation of composite material, EDS analysis were performed (Figure 3d). EDS MAP plots indicate the presence of C, Ag, S, O, and Na in CS-LS/AgNPs and their uniform distribution (Figure 3e–i). In summary, the EDS results demonstrate the successful combination of LS with CS and the successful preparation of nanosilver. Furthermore, the porous properties including the specific surface area (SSA) and average pore size (APS) of CS-LS/AgNPs were calculated to be 0.93 m^2^/g and 2.95 nm, respectively, from the N_2_ sorption–desorption isotherm (Figure 3i).

**Figure 4:**
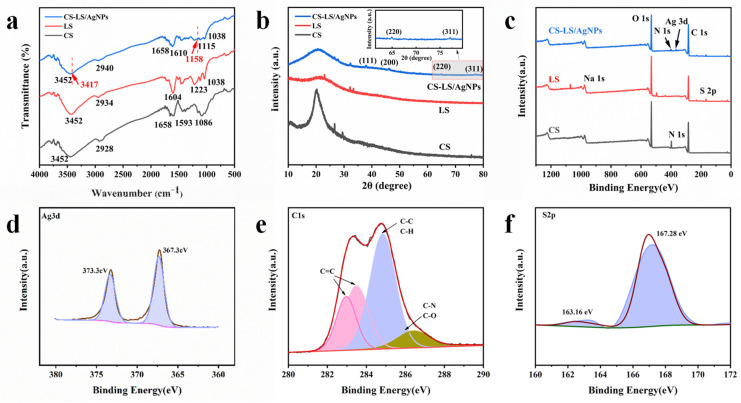
(**a**) FTIR, (**b**)XRD, (**c**) XPS survey of CS-LS/AgNPs, and XPS high-resolution pattern of (**d**) Ag 3d, (**e**) C 1s, and (**f**) S 2p.

FT-IR spectra of CS, CS-LS, and CS-LS/AgNPs are shown in Figure 4a. In the spectra of CS, the broad peaks at 3452 cm^−1^ are attributed to the stretching vibrations of O-H and N-H bonds. The peaks at 2934 cm^−1^ are assigned to the asymmetric vibrations of C-H bonds. The absorption peaks at 1658 cm^−1^ and 1593 cm^−1^ are attributed to the characteristic bands of amide I and amide II of CS, respectively [24,25,26]. After preparing CS and LS into granular composites (CS-LS), the stretching peak of amide I band of CS shifted from 1658 cm^−1^ to 1604 cm^−1^, indicating the presence of hydrogen bonds between CS and LS. As for the characteristic peaks of LS, the peaks at 3252 cm^−1^ and 2934 cm^−1^ correspond to the stretching vibration of -OH and asymmetric stretching vibration of -CH, respectively, and the peak at 1046 cm^−1^ is derived from S=O symmetric stretching of the -SO_3_ group) [27,28]. In the FTIR spectrum of CS-LS/AgNPs, it can be seen that, compared with CS-LS, two peaks are generated at 3417 cm^−1^ and 1158 cm^−1^, corresponding to the characteristic peaks of AgNPs [29]. As shown in Figure 4b, in the XRD pattern of CS-LS/AgNPs, apart from the original characteristic peaks of CS and LS, four characteristic peaks at 2θ = 37.53°, 46.35°, 64.94°, and 77.69° are attributed to the (111), (200), (220), and (311) planes of face-centered cubic silver, respectively. The peak corresponding to the (111) plane is stronger than those in other planes, indicating the dominance of the (111) plane [30,31]. In order to further determine the changes in element distribution and electron density, XPS measurements were performed on the prepared CS-LS/AgNPs. As shown in Figure 4c, it is revealed that the prepared photocatalyst includes C, S, and Ag elements. From the narrow scan spectrum of Ag 3d (Figure 4d), it can be seen that the two peaks of 367.3 eV and 373.3 eV are Ag 3d_5/2_ and Ag 3d_3/2_, respectively, which are typical binding energies of metallic Ag, indicating that Ag nanoparticles are formed on the surface of CS-LS. The high resolution of C 1s signal (Figure 4e) detected four signals at 282.8 eV, 283.6 eV, 284.8 eV, and 286.5 eV, with spikes at 284.8 eV attributed to C-C and C-H bonds in CS and LS. The peak of binding energy at 282 eV~284 eV is assigned to C=C [24]. The peak at 286.5 eV is attributed to C-N, C-O in CS, and C-O in LS [32]. For S 2p, the main peak of 167.28 eV is attributed to S=O of the -SO_3_ group in LS (Figure 4f). The above results proved that CS-LS formed a relatively strong crosslinked composite material, and the silver nanoparticles were reduced in situ through the reducing groups on the LS surface and successfully loaded onto the surface of particles to form CS-LS/AgNPs composite materials.

### 3.2. Catalytic Degradation Performance

**Figure 5:**
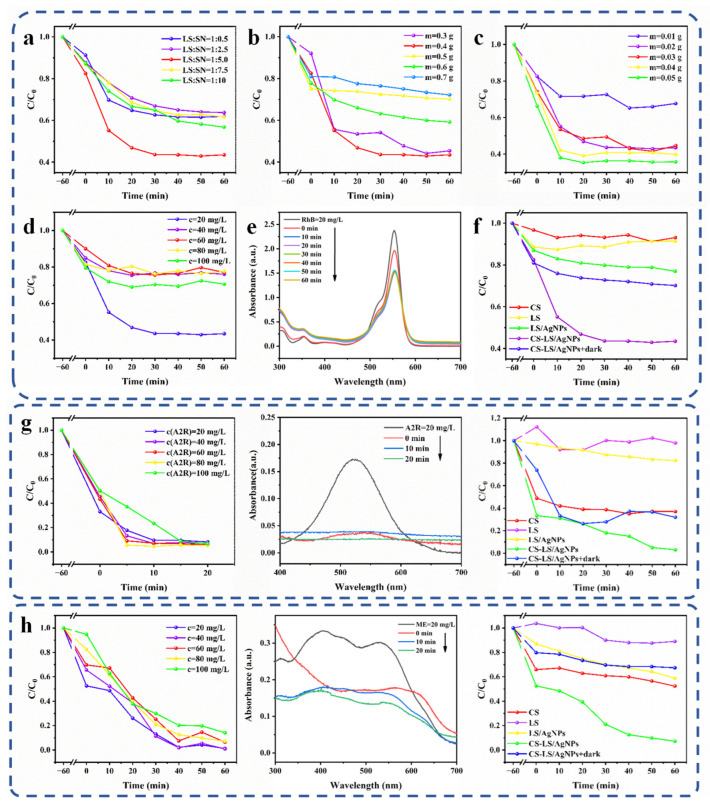
(**a**–**f**) Effect of different factors on RhB decoloration in CS-LS/AgNPs, degradation percentage of CS-LS/AgNPs on (**g**) Telon Red A2R and (**h**) Direct Dark Brown ME.

The enhanced photoadsorption activity of CS-LS/AgNPs composites is mainly due to the adsorption of dyes by the CS-LS layer and the catalytic degradation of dyes by ·O_2_^−^ and ·OH produced by silver nanoparticles in RhB under light irradiation, as well as by electron–hole pairs. In order to distinguish the effect of prepared CS-LS/AgNPs on dye adsorption and photocatalysis, the adsorption equilibrium was achieved under dark conditions for 1 h before each experiment, and then the photocatalysis experiment was carried out. The effect of LS: SN ratio on the decolorization effect of RhB was studied first. Briefly, 20 mg of prepared CS-LS/AgNPs were added to 20 mL of RhB (20 mg/L) solution. As shown in Figure 5a, the removal rate of RhB increases gradually with the reaction time. Moreover, with the increase of LS: SN, the effect of the composite on the removal rate of RhB varies, mainly due to the large surface area of LS, which can adsorb dyes to a large extent. However, the excessive loading of LS reduces the proportion of nanosilver, thus affecting the photoresponse of AgNPs. It was found that when LS: SN reaches 1:5, the degradation of RhB by prepared CS-LS/AgNPs can reach 55% within 30 min. On the basis of determining the optimal ratio of LS: SN (1:5), the catalytic degradation experiments were carried out under different conditions. The effect of CS content on RhB degradation was also investigated. As shown in Figure 5b, the removal rate of RhB increases gradually with the reaction time. With the increase in CS content, dye removal first increases and then gradually decreases, indicating that excessive CS loading will affect the dye removal rate of the whole composite material. Therefore, considering the efficiency and economic benefits, 0.3 g of chitosan was chosen to be used in the following experiments. Subsequently, the effects of CS-LS/AgNPs content, dye concentration, decolorization time, and blank control of different materials on RhB decoloration were performed (Figure 5c–f). From Figure 5c, it can be seen that when the content of CS-LS/AgNPs was increased from 0.2 g to 0.4 g, the dye removal did not improve greatly. Thus, 20 mg of CS-LS/AgNPs is suitable for dye degradation. The dye concentration is also an unstable parameter of color removal rate, which affects the effectiveness of the removal process. As shown in Figure 5d,e, the results show that the dye removal rate is affected by the concentration of dye solution. At the initial concentration of 20 mg/L, the removal rate of the dye is the highest, and at a specific reaction time, the removal rate will decrease as the dye concentration increases. However, for the maximum concentration (100 mg/L) of dye solution, the removal rate remained above 30%. In addition, it can be found that at higher dye concentration, the dye removal rate constant at the beginning stage of adsorption is generally greater than that at low concentration, which may be due to the greater contact probability between CS-LS/AgNPs and dyes at higher concentration. When the dye concentration is low, the dye can form a stable reaction system with the photogenerated electron holes more quickly, and the catalytic efficiency is higher, resulting in a relatively high dye removal rate. Furthermore, as shown in Figure 5f, we explored the photocatalytic degradation percentage of dyes by CS, LS, LS/AgNPs, CS-LS/AgNPs, and “CS-LS/AgNPs + dark”. It can be seen that CS-LS/AgNPs play a distinct role in dyes degradation, and photocatalysis is vital.

### 3.3. Applicability of CS-LS/AgNPs to a Variety of Dyes

In order to investigate the decolorization effects of prepared CS-LS/AgNPs on different dyes, Telon Red A2R and Direct Dark Brown ME were also used as model dyes. Figure 5g,h show the degradation result of CS-LS/AgNPs on the two dyes. It can be seen that CS-LS/AgNPs have ideal degradation rates for Telon Red A2R and Direct Dark Brown ME, which are 95% and 97%, respectively. It is proved that CS-LS/AgNPs are stable in photocatalytic adsorption and have certain universality in photocatalytic degradation of dyes with different structures. It should be explained that when LS alone is used for dye degradation, the LS powder may be mixed during the absorbance measurement, resulting in a high dye concentration and biasing the measurement results.

### 3.4. Kinetics Research

In order to further study the catalytic activity of CS-LS/AgNPs composites, decolorization experiments were carried out under different conditions. Since the decolorization of dyes may be affected by different factors, the decolorization types of dyes are discussed. The corresponding kinetic equation can be described as follows:

Pseudo-first-order kinetic equation: ln (C/C_0_) = −k_1_t (1)

**Figure 6:**
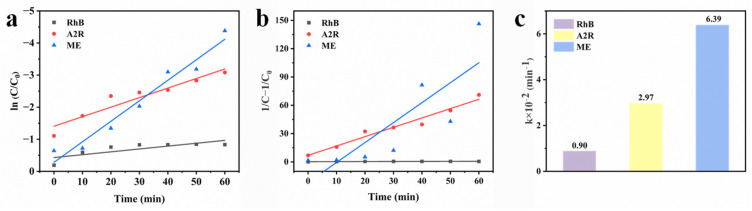
(**a**,**b**) Linear fittings of pseudo-first-order and pseudo-second-order kinetics; (**c**) comparisons of the corresponding apparent rate constants for the degradation of RhB, A2R, and ME.

Pseudo-second-order kinetic equation: 1/C − 1/C_0_ = k_2_t (2)

where C_0_ is the original dye concentration, C is the dye concentration at time t in the presence of CS-LS/AgNPs, and k_1_ and k_2_ are rate constants. As shown in Figure 6, it is obvious that the linear regression of CS-LS/AgNPs composites agrees well with the pseudo-first-order kinetic model. In addition, the rate constants of the degradation of three dyes by CS-LS/AgNPs were also studied. It is evident that there is a significant difference in the degradation of the composites for different dyes, where the rate constants of ME and A2R are six and three times higher than RhB, respectively.

### 3.5. Reusability of CS-LS/AgNPs

**Figure 7:**
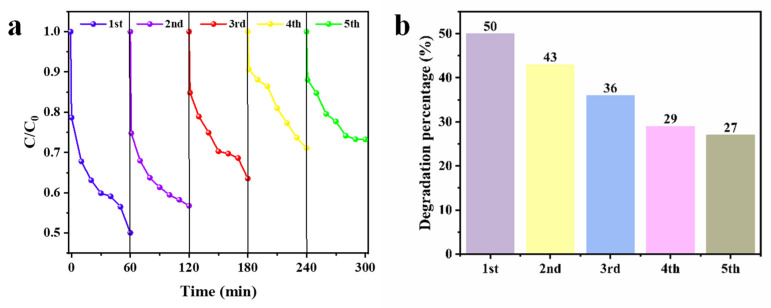
Cyclic stability of CS-LS/AgNPs for RhB dye decolorization: (**a**) Dye decolorisation process per cycle and (**b**) final degradation percentage on RhB dye after each cycle.

Figure 7 shows the reusability of the CS-LS/AgNPs composite for RhB dye decolorization. After repeated use for five cycles, the catalytic decolorization performance slightly decreases with the increase in running times, but the reused catalyst still shows usable activity, removing 27% of RhB within one hour on the fifth cycle.

### 3.6. Photoadsorption Mechanism

**Figure 8:**
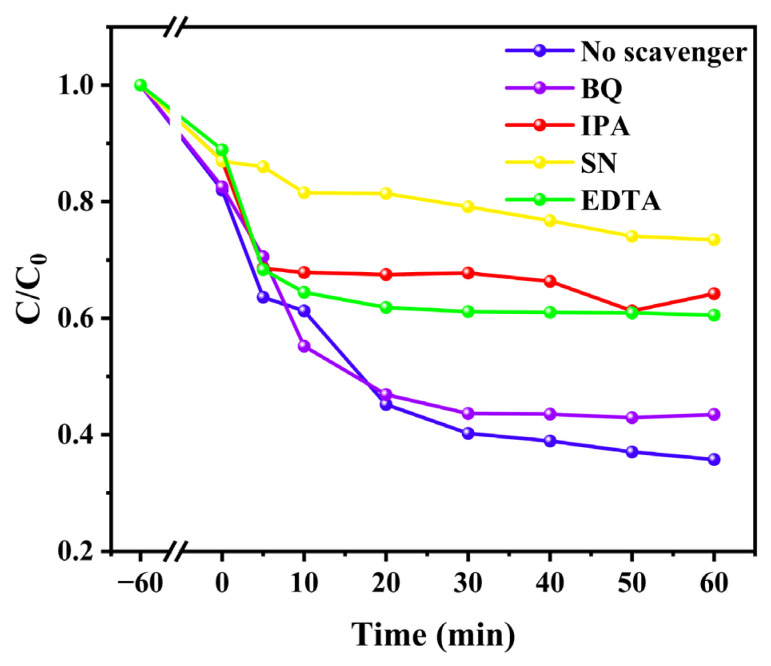
Effects of scavengers on the photodegradation of RhB over CS-LS/AgNPs.

The potential mechanism and pathway of dye removal by CS-LS/AgNPs composites was proposed in this section. First, in order to explore the active groups under CS-LS/AgNPs photocatalytic conditions, IPA, EDTA, AgNO_3_, and BQ were used as scavengers of ·OH, h^+^, ·O_2_^−^, and e^−^ under ultraviolet irradiation (Figure 8). Based on the results of the capture experiment, a possible reaction process is proposed [30,33], as follows:

AgNPs + hv + UV → e^−^ + h^+^ (3)

O_2_ + e^−^ → ·O_2_^−^ (4)

h^+^ + OH^−^ → ·OH (5)

·O_2_^−^ (h^+^/·OH) + RhB → H_2_O + CO_2_ + other degradation products (6)

**Figure 9:**
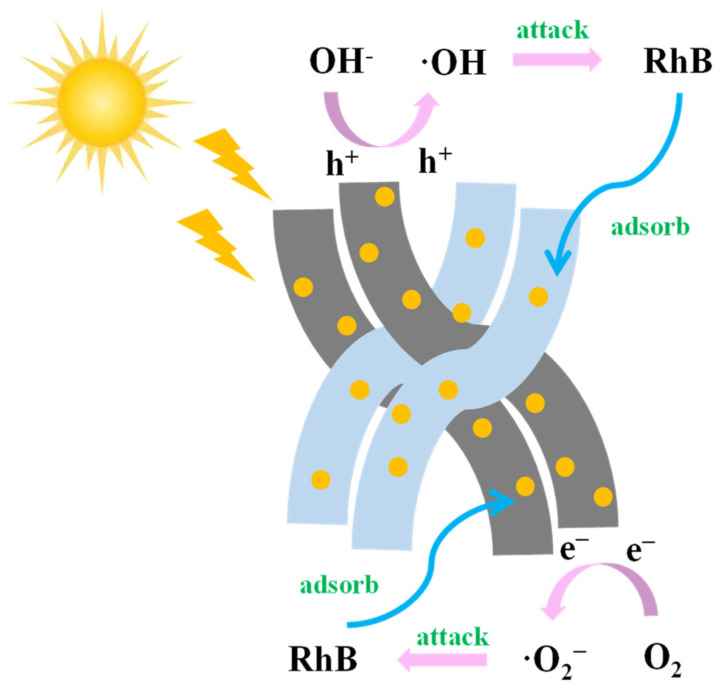
Potential mechanism of CS-LS/AgNPs photoadsorption.

From the above results, the photocatalytic mechanism of CS-LS/AgNPs is proposed (Figure 9). The photocatalytic degradation mechanism mainly consists of three steps: (a) the adsorption of pollutants/dyes on the surface of the composite, (b) the absorption of light by the composite, and (c) the generation of e^−^–h^+^. Photoinduced electron–hole pairs are essentially responsible for generating the most active free radicals for efficient photocatalysis [34].

## 4. Conclusions

In conclusion, a novel CS-LS/AgNPs composite material was synthesized. During the synthesis process, Ag nanoparticles were reduced in situ and loaded onto the CS-LS surface. The cross-linking between CS and LS provided considerable stability for AgNPs. The prepared composite material has ideal degradation percentage for Telon Red A2R and Direct Dark Brown ME, which are 95% and 97%, respectively, although it just has 55% of degradation percentage in photocatalytic degradation of RhB. The CS-LS/AgNPs still show usable activity after five cycles of experiments. The prepared composite material is suitable for the removal of various dyes, which provides the possibility for the treatment of dye wastewater by biobased composite.

## Data Availability

Data are contained within the article.
